# A new role for Holliday junction resolvase Yen1 in processing DNA replication intermediates exposes Dna2 as an accessory replicative helicase

**DOI:** 10.15698/mic2017.01.554

**Published:** 2017-01-02

**Authors:** Benoît Falquet, Ulrich Rass

**Affiliations:** 1 Friedrich Miescher Institute for Biomedical Research, Maulbeerstrasse 66, CH-4058 Basel, Switzerland.; 2 University of Basel, Petersplatz 10, CH-4003 Basel, Switzerland.

**Keywords:** replication stress, DNA repair, genome stability, replication fork restart, anaphase bridges, chromosome non-disjunction, structure-specific nucleases

## Abstract

DNA replication is mediated by a multi-protein complex known as the replisome. With the hexameric MCM (minichromosome maintenance) replicative helicase at its core, the replisome splits the parental DNA strands, forming replication forks (RFs), where it catalyses coupled leading and lagging strand DNA synthesis. While replication is a highly effective process, intrinsic and oncogene-induced replication stress impedes the progression of replisomes along chromosomes. As a consequence, RFs stall, arrest, and collapse, jeopardizing genome stability. In these instances, accessory fork progression and repair factors, orchestrated by the replication checkpoint, promote RF recovery, ensuring the chromosomes are fully replicated and can be safely segregated at cell division. Homologous recombination (HR) proteins play key roles in negotiating replication stress, binding at stalled RFs and shielding them from inappropriate processing. In addition, HR-mediated strand exchange reactions restart stalled or collapsed RFs and mediate error-free post-replicative repair. DNA transactions at stalled RFs further involve various DNA editing factors, notably helicases and nucleases. A study by Ölmezer *et al*. (2016) has recently identified a role for the structure-specific nuclease Yen1 (GEN1 in human) in the resolution of dead-end DNA replication intermediates after RF arrest. This new function of Yen1 is distinct from its previously known role as a Holliday junction resolvase, mediating the removal of branched HR intermediates, and it becomes essential for viable chromosome segregation in cells with a defective Dna2 helicase. These findings have revealed greater complexity in the tasks mediated by Yen1 and expose a replicative role for the elusive helicase activity of the conserved Dna2 nuclease-helicase.

The Dna2 nuclease-helicase has emerged as a multifunctional mediator of genome stability. Other labs have shown that Dna2’s nuclease activity is involved in DNA end-resection, facilitating HR-mediated DNA double-strand break repair and the resetting of reversed RFs. The Dna2 helicase activity appears to be dispensable for these processes and its function *in vivo* has remained enigmatic. In *Saccharomyces cerevisiae*, cells harboring various different point mutations within the Dna2 helicase domain share a common sensitivity to the DNA alkylating agent methyl methane sulfonate (MMS). Ölmezer *et al*. (2016) now provide evidence that this phenotype relates to a critical role of the Dna2 helicase at stalled RFs. Key for elucidating this function was a synthetic sick phenotype that arises when the Holliday junction resolvase *YEN1* is deleted in cells expressing Dna2 R1253Q. Ölmezer and co-workers first demonstrated that Dna2 R1253Q, encoded by mutant allele *dna2-2 *and bearing the R to Q amino acid substitution in an ATP binding loop between the characteristic RecA lobes of the Dna2 superfamily 1 helicase domain, is indeed helicase-dead but retains full nuclease activity. Cells expressing Dna2 R1253Q exhibit chronic checkpoint activation and a delay in G2/M phase of the cell cycle. Nevertheless, cell viability remained high in Dna2 R1253Q cells, dropping significantly when *YEN1* was deleted, while the kinetics of bulk DNA synthesis during S phase were indistinguishable from wild-type in Dna2 R1253Q cells in the presence or absence of Yen1.

Analysis during a single cell cycle showed that Dna2 R1253Q cells exhibit an unusual, biphasic checkpoint activation pattern in response to mild, acute replication stress induced through nucleotide depletion by hydroxyurea (HU). Like wild-type cells, Dna2 R1253Q and Dna2 R1253Q *yen1*∆ cells activated the replication checkpoint in the presence of HU, followed by checkpoint silencing and completion of bulk DNA synthesis after removal of the drug. However, in contrast to wild-type cells, Dna2 helicase-defective cells failed to divide, and instead exhibited reemerging checkpoint signaling, elicited by the G2/M DNA damage checkpoint. Yen1 was unable to suppress this unscheduled G2/M checkpoint activation. This may seem counter-intuitive, given that Yen1 is the factor maintaining viability in Dna2 helicase-defective cells. However, considering that Yen1 is tightly controlled, so that accumulation within the nucleus occurs only after cells make the G2-M transition and enter anaphase, the inability to prevent Dna2-related DNA lesions during the course of S and G2/M phase is perhaps not surprising. Indeed, Yen1 remained cytoplasmic in Dna2 R1253Q cells for extended periods of time while the G2/M arrest was maintained. When cells made the transition into M phase after experiencing replication stress, Yen1 became nuclear and cell survival was then fully dependent upon the nuclease activity of Yen1, which suppressed toxic chromosome entanglements detected as anaphase bridges. One implication of these findings is that Dna2 helicase activity is needed at stalled RFs, ensuring that the genome is fully replicated and chromosomes are no longer attached to one another at segregation.

DNA synthesis is not globally affected in Dna2 R1253Q cells, suggesting the Dna2 helicase is only needed at a subset of troubled RFs to promote replication restart or fork stability, helping to ensure full genome duplication either by reinitiating DNA synthesis or by maintaining arrested forks in a conformation favorable for subsequent fork fusion with an oncoming, active fork (Figure 1; pathways indicated by green arrows). A role in replication termination is consistent with an accumulation of converged yet unresolved RFs in Dna2 helicase-defective cells, which were detected by two-dimensional gel electrophoresis of replication intermediates at the natural replication fork pausing site in the rDNA. Further clues as to the actions of Dna2 come from the biphasic checkpoint activation pattern seen in Dna2 R1253Q cells upon exposure to acute replication stress. The gap in checkpoint signaling that was observed after replication checkpoint silencing and before DNA damage checkpoint activation suggests that replication intermediates that escape the attention of Dna2 are not at first detected by the DNA damage checkpoint. This could be explained if the sensitivity of the DNA damage checkpoint increases over time such that initially checkpoint-blind replicative lesions can be detected as cells approach G2/M phase. We favor a different explanation, namely that an initial checkpoint-blind DNA structure is subsequently converted into a detectable lesion, and that Dna2 either prevents or counteracts this process. An attractive possibility is that fork reversal is involved, a well-described phenomenon in response to replication stress that entails annealing of the nascent DNA strands. As a result, three-way RFs are converted into a so-called chicken foot structure with four DNA branches similar to a HJ. Importantly, the tip of the newly extruded branch is indistinguishable from a DNA double-strand break, a structure that elicits a strong DNA damage checkpoint response (Figure 1). RF conversion into a chicken foot intermediate is also consistent with the unique requirement for Yen1 in Dna2 helicase-defective cells. Yen1 possesses the rare ability to recognize and resolve DNA four-way junctions through coordinated incisions on either side of the branch point and is therefore perfectly suited to remove dead-end fork-reversal intermediates upon its activation in anaphase, just prior to chromosome segregation. Consistent with this notion, Ölmezer *at al*. (2016) showed, for the first time, that Yen1 is capable of removing persistent dead-end replication intermediates - detected by two-dimensional gel electrophoresis - in Dna2 helicase-defective cells. Yen1 is thus a versatile mitotic DNA de-branching nuclease whose actions are not restricted to canonical Holliday junction resolution downstream of HR-mediated strand exchange. Given the specificity of *DNA2*’s genetic interaction with *YEN1*, which was found not to extend to the other Holliday junction resolvases *MUS81-MMS4* and *SLX1-SLX4*, Yen1’s activity towards dead-end replication intermediates, as opposed to HR structures, appears most relevant to maintain viability in Dna2 helicase-defective cells.

**Figure 1:**
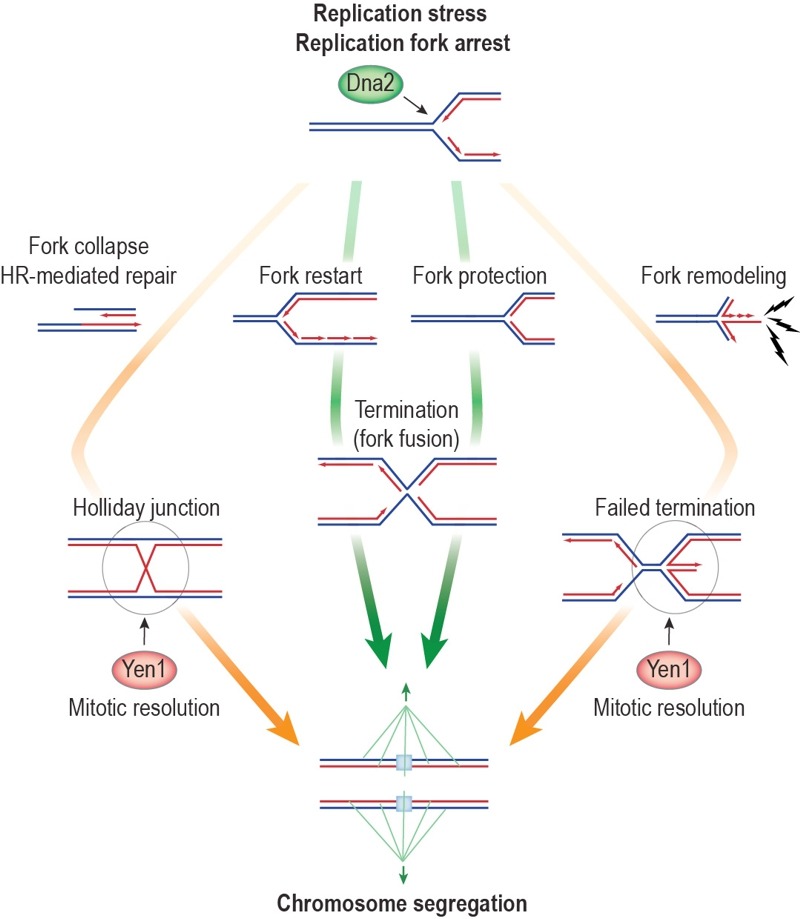
FIGURE 1: Dna2 helicase activity promotes the recovery of stalled DNA replication intermediates. Dna2 acts as an accessory replicative helicase, suppressing toxic replication intermediates and chromosome non-disjunction after RF arrest. This suggests that the Dna2 helicase activity facilitates fork restart or fork protection (green arrows), promoting full genome replication and/or fork fusion during replication termination. Arrested RFs that escape the attention of Dna2 may collapse, triggering HR-mediated restart. Alternatively, unscheduled fork remodeling may occur with the resulting chicken foot structure constituting a potential source for DNA damage checkpoint signaling (lightning bolts) in Dna2 helicase-defective cells, and a possible obstruction to replication termination. These pathways (orange arrows) lead to chromosome entanglements that are resolved by Yen1 in anaphase, ensuring viable chromosome segregation.

This is consistent with the finding that the requirement for Yen1 in Dna2 helicase-defective cells cannot be suppressed by eliminating HR. Nonetheless, we expect that Yen1 (in this case redundantly with Mus81-Mms4) contributes to the resolution of HR intermediates in Dna2 helicase-defective cells, which exhibit elevated levels of HR, likely due to compensatory RF recovery by HR and/or HR-dependent repair of Yen1-induced DNA breaks at troubled RFs. Indeed, the repair events that take place downstream of Yen1-mediated resolution of dead-end replication intermediates have not been addressed and remain to be elucidated. It is remarkable that the cleavage of post-replicative sister chromatid entanglements by Yen1 in anaphase does not appear to interfere with mitosis. Ölmezer and co-workers showed that disruption of the G2/M checkpoint restored normal mitotic progression and full viability in Dna2 R1253Q cells after acute replication stress, provided Yen1 was functional. This suggests that Yen1-dependent repair is either straightforward and simple enough to be completed in anaphase, or that Yen1-dependent repair intermediates can be safely transmitted to daughter cells for processing in the next cell cycle. Either way, Yen1 provides efficient protection against anaphase bridges and mitotic catastrophe; it will be interesting to assess whether there is a price to pay for this last-minute intervention by Yen1 with regard to genetic stability.

A key point of the study by Ölmezer *et al. *(2016) is that it demonstrates how intimately the Dna2 helicase activity is linked to replication. It is worth pointing out that loss of Yen1, which in itself does not lead to any overt phenotype, strongly compromises growth in Dna2 helicase-defective cells, indicating that post-replicative chromosome entanglements and chromosome non-disjunction occur even in unperturbed conditions. This highlights the so-far underestimated importance of the Dna2 helicase in ameliorating the consequences of endogenous RF stalling and avoiding underreplication. We think the Dna2 helicase is best described as an accessory replicative helicase.

Replication stress and RF demise is an important driver of genome instability and cancer formation. A better understanding of the molecular choreography at stalled RFs therefore has direct biomedical implications. *DNA2* has been linked to a number of human disease syndromes and is frequently overexpressed in cancer, suggesting that cancer cells may use the activities of DNA2 to overcome excessive levels of RF stalling. Inhibiting the DNA2 helicase could in principle provide a therapeutic avenue aimed at killing cancer cells by stress-overload.

